# Identification of Microbial Communities in Open and Closed Circuit Bioelectrochemical MBRs by High-Throughput 454 Pyrosequencing

**DOI:** 10.1371/journal.pone.0093842

**Published:** 2014-04-04

**Authors:** Jian Huang, Zhiwei Wang, Chaowei Zhu, Jinxing Ma, Xingran Zhang, Zhichao Wu

**Affiliations:** 1 State Key Laboratory of Pollution Control and Resource Reuse, School of Environmental Science and Engineering, Tongji University, Shanghai, P. R. China; 2 Chinese Research Academy of Environmental Sciences, Beijing, P. R. China; Wageningen University, Netherlands

## Abstract

Two bioelectrochemical membrane bioreactors (MBRs) developed by integrating microbial fuel cell and MBR technology were operated under closed-circuit and open-circuit modes, and high-throughput 454 pyrosequencing was used to investigate the effects of the power generation on the microbial community of bio-anode and bio-cathode. Microbes on the anode under open-circuit operation (A_O_) were enriched and highly diverse when compared to those on the anode under closed-circuit operation (A_C_). However, among the cathodes the closed-circuit mode (C_C_) had richer and more diverse microbial community compared to the cathode under open-circuit mode (C_O_). On the anodes A_O_ and A_C_, *Proteobacteria* and *Bacteroidetes* were the dominant phyla, while *Firmicutes* was enriched only on A_C_. *Deltaproteobacteria* affiliated to *Proteobacteria* were also more abundant on A_C_ than A_O_. Furthermore, the relative abundance of *Desulfuromonas*, which are well-known electrogenic bacteria, were much higher on A_C_ (10.2%) when compared to A_O_ (0.11%), indicating that closed-circuit operation was more conducive for the growth of electrogenic bacteria on the anodes. On the cathodes, *Protebacteria* was robust on C_C_ while *Bacteroidetes* was more abundant on C_O_. *Rhodobacter* and *Hydrogenophaga* were also enriched on C_C_ than C_O_, suggesting that these genera play a role in electron transfer from the cathode surface to the terminal electron acceptors in the bioelectrochemical MBR under closed-circuit operation.

## Introduction

Microbial fuel cells (MFCs) offer a promising technology for both organic waste treatment and simultaneous power generation, and have attracted attention in the past decade. However, the practical applications of MFCs are limited by disadvantages such as low treatment efficiency and poor effluent quality. Membrane bioreactor (MBR) is another promising wastewater treatment process that has achieved great advances in the past two decades. MBR is considered as an alternate technology for conventional activated sludge (CAS) systems due to its high treatment efficiency and good effluent quality [1]. However, its wide-spread application is hindered by the high energy consumption.

Recently, efforts have been made to integrate MFC and MBR for wastewater treatment. It is presumed that the power generated by MFC from wastewater will partially offset the energy requirement of MBR, which in turn could increase treatment efficiency and enhance effluent quality of MFC. In a recently developed MFC-MBR system the aeration tank of an MBR was used as the cathode chamber [2]. Stainless-steel mesh membrane module has also been used as the cathode in an MFC-MBR system, which achieved a maximum power density of 8.62 W/m^3^ [3]. In another study, removal of 92.4% chemical oxygen demand (COD) and 4.35 W/m^3^ power density has also been reported [4]. These studies demonstrate the potential of bioelectrochemical MBR created by MFC-MBR integration for simultaneous wastewater treatment and energy production.

In these integrated systems, microorganisms play critical roles in both power generation and also wastewater treatment. Power is generated when microorganisms on the anode act as catalysts to oxidize organic matters and transfer the generated electrons to the cathode though an external circuit while the microbes on the cathode function as biocatalysts and accept electrons. On the other hand, biological wastewater treatment is generally carried out in the cathode chamber (aerobic MBR basin), which also impacts the microbial community on the cathode. A number of studies have determined the microbial community in typical MFCs [5]–[7]. However, studies on the microbial communities in bioelectrochemical MBRs are limited. Due to the distinct differences between typical MFCs and integrated MFC-MBR systems, the microbes in these newer systems are expected to be unique. Therefore, understanding their composition and functions will allow optimization of these integrated systems.

The goal of this study was to characterize the microbial community in the integrated MFC-MBR systems. To achieve this goal, we developed two identical bioelectrochemical membrane bioreactors (MBRs) by integrating MFC and MBR technology and operated them under closed-circuit and open-circuit modes. We then compared the microbial communities between the two MBRs using high-throughput 454 pyrosequencing to investigate the effects of power generation on microbial community changes on the bio-anode and bio-cathode. The results of this study provide insights into the microbial communities and their functions in MFC-MBR systems, and will facilitate optimization of these integrated systems for efficient wastewater treatment and power generation.

## Materials and Methods

### Experimental set-up

**Figure 1:**
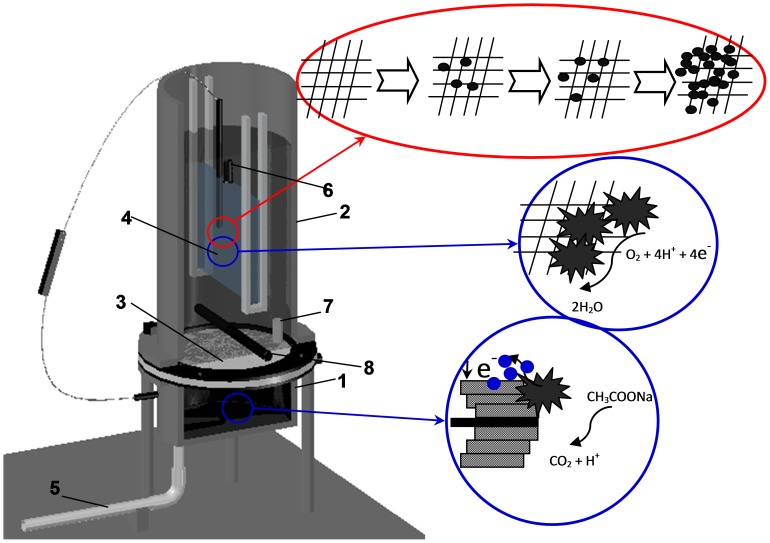
Schematic diagram of the bioelectrochemical MBR system. (1) Anode chamber; (2) cathode chamber; (3) cloth separating the two chambers; (4) stainless-steel mesh membrane module; (5) influent pipe; (6) effluent pipe; (7) connecting pipe; (8) air diffuser.

The schematic diagram of the experimental setup is shown in Fig. 1. The bioelectrochemical MBR was designed to have two cylinder compartments both with the same inner diameter of 10 cm, and designated as the anode and cathode chambers. The two chambers were separated by a cloth pretreated with polyvinylidene fluoride. A 3-cm long Plexiglas tube with 8 mm diameter was installed in the anode chamber to enable continuous upflow of wastewater from anode to the cathode.

The anode chamber was filled with graphite felts (10-cm diameter) that were cut into pieces of 1 cm×1 cm (width × thickness). Total volume of the anode chamber was 140 mL and the volume of water decreased to 37 mL after the installation of graphite felts. Then, a graphite rod (6 mm diameter) was inserted in the anode graphite felts and connected to the external circuit. The cathode was a flat-sheet membrane (4 cm×8 cm) made of stainless mesh (pore size 48 μm), and was installed in the cathode chamber with an effective volume of 1.25 L. Two graphite rods were fastened on each side of the membrane module using stainless steel wires. Two perforated Plexiglas tubes were mounted below the membrane module to supply oxygen and mix liquids with an aeration intensity of 0.67 L/min.

In the bioelectrochemical MBR under closed-circuit operation (MBR_C_), the graphite rods in both anode and cathode were connected with copper wires with 470 or 100 Ω external resistance, while the graphite rods remained open in the MBR under open-circuit operation (MBR_O_), which also served as a control reactor. This design enabled us to compare the differences in the microbial communities between the two systems, and analyze the impact of different modes of power generation on variations in the microbial communities.

### Inoculation and operation

The anode chambers in both bioelectrochemical MBRs were inoculated with 10 mL sludge obtained from the anaerobic unit of an anaerobic/anoxic/oxic-MBR system, which is documented in a previous publication [8]. The mixed liquor suspended solid (MLSS) and mixed liquor volatile suspended solid (MLVSS) concentrations of the inoculated sludge were 16.2 g/L and 9.0 g/L, respectively. The cathode chambers were seeded with activated sludge from Shanghai Quyang municipal wastewater treatment plant in China, which had an MLSS concentration of 3.9 g/L and an MLVSS concentration of 2.8 g/L.

After inoculation, synthetic wastewater was fed into the anode chamber, and then allowed to flow into the cathode chamber. Composition of the synthetic wastewater was: CH_3_COONa·3H_2_O 640 mg/L, NH_4_Cl 57 mg/L, K_2_HPO_4_·3H_2_O 22 mg/L, CaCl_2_ 11.5 mg/L, MgSO_4_ 12 mg/L, and 10 mL of the trace element solution, which is similar to those used in previous studies [3], [9]. Both reactors were placed at room temperature (25±1°C) and sludge was not discharged during the operation.

At the end of the experimental period (75 days), biofilms were scraped from MBR_C_ anode (A_C_), MBR_C_ cathode (C_C_), MBR_O_ anode (A_O_) and MBR_O_ cathode (C_O_) to analyze the differences in the microbial communities using 454 pyrosequencing.

### Analysis of the microbial communities

#### DNA extraction and PCR amplification

DNA was extracted from the microbial communities in A_C_, A_O_, C_C_ and C_O_ according to methods described previously [10]. To amplify the 16S rRNA from the samples the following universal primers were used: 8F (5′-AGAGTTTGATCCTGGCTCAG-3′) and 533R (5′-TTACCGCGGCTGCTGGCAC-3′). Each PCR reaction was carried out in a 20 μL reaction volume containing 4 μL 5× FastPfu Buffer, 2 μL 2.5 mM dNTPs, 0.4 μL of each primer, 0.5 μL DNA and 0.4 μL FasrPfu Polymerase (TransStart FastPfu DNA Polymerase, TransGen, China). The thermocycling steps used for the PCR were: 95°C for 2 min, 25 cycles of 95°C for 30 sec followed by 55°C for 30 sec, and 72°C for 30 sec, and a final extension at 72°C for 5 min. After amplification, the amplicons were purified directly from the PRP mixture using the UNIQ-10 PCR purification Kit (Sangon, Shanghai, China) and quantified using TBS-380 (Turner BioSystems, Inc., USA).

#### 454 pyrosequencing

A mixture of the purified amplicons was used for 454 pyrosequencing on a Roche massively parallel 454 GS-FLX Titanium sequencer (Roche 454 Life Sciences, Branford, CT, USA). To improve the quality of pyrosequencing data, defective reads were removed from the libraries; these included reads without a recognizable reverse primer, reads shorter than 150 bp, and those containing any ambiguous base call [10]. Then barcodes and primers were trimmed from the resulting sequences. The final pyrosequencing data contained 6381 (A_O_), 6854 (A_C_), 12243 (C_O_), 7097 (C_C_) high quality V1–V3 tags for the 16S rRNA-gene. The raw reads were deposited into the NCBI Sequence Read Archive (SRA) database (Accession Number: SRA114114).

#### Classification of the microbial communities

Pyrosequencing reads were clustered into operational taxonomic units (OTUs) with an average length of 390 bp. Then the OTUs were further clustered using the MOTHUR program (http://www.Mothur.org/wiki/Main-Page) with sequence distances set at 0.03 or 0.05. Based on these clusters the following parameters were calculated for each sample: Shannon diversity index (http://www.mothur.org/wiki/Shannon), Chao1 richness (http://www.mothur.org/wiki/Chao), abundance-based coverage using the abundance coverage estimator (ACE) (http://www.mothur.org/wiki/Ace), Good's coverage (http://www.mothur.org/wiki/Coverage), and the rarefaction curves at α 0.03 and 0.05. Based on MOTHUR and SILVA106 database, representative reads from the clusters were classified with a confidence threshold of 80% at the levels of both phyla and genera.

## Results and Discussion

### Performance of the bioelectrochemical MBRs

In the bioelectrochemical MBR under closed-circuit operation, the stainless-steel membrane module with biofilm formation not only serves as a biocathode for the MFC but also as a dynamic membrane for efficient solid/liquid separation [4]. In both MBR systems, effluent turbidity of 0.8 NTU (Nephelometric turbidity unit) was reached within 24 h. In addition, the average efficiencies of chemical oxygen demand (COD) and NH_4_^+^-N removal during the operation were 86.1% and 97.5% for MBR_C_, and 84.9% and 96.4% for MBR_O_. Maximum power density of the MBR under closed-circuit operation was as high as 8.6 W/(m^3^ anode net volume), indicating that the biochemical MBR not only achieved efficient wastewater treatment but also power production [4]. Experimental results also demonstrated that the MFC (the anodic chamber under closed-circuit operation) could increase the degradation rate of organic matters by 7.0–13.0% compared with the anode chamber in the open-circuit mode, and the Coulombic efficiencies of the MFC under closed-circuit operation were 1.9%, 3.2%, 4.5% and 12.3% in the four runs, respectively [4].

### Microbial richness and diversity

In our study, four 16S rDNA gene libraries were constructed by high-throughput sequencing of microbial communities from A_O_, A_C_, C_O_ and C_C_ samples. After trimming, sorting and quality control, 6381(A_O_), 6854(A_C_), 12243(C_O_) and 7097(C_C_) high-quality sequence tags (average length - 390 bp) were clustered into 1859 (A_O_), 1488 (A_C_), 1997 (C_O_) and 1856 (C_C_) operational taxonomic units (OTUs) at 3% distance thresholds, and 1602 (A_O_), 1211 (A_C_), 1583 (C_O_) and 1493 (C_C_) OTUs at 5% distance thresholds (Table 1). The abundance-based coverage estimator (ACE), Chao1, Shannon and Good Coverage are also presented in Table 1. Higher numbers of OTUs were estimated for A_O_ sample (4719 and 7816 in A_O_, 3424 and 5712 in A_C_, respectively) with infinite sampling at 3% distance by Chao1 estimator and ACE. This indicated that the richness of the bacterial communities in the MFC anode operated under open circuit was higher than that under closed circuit mode. However, for the cathode samples, microbial richness of Cc was greater than Co (Table 1). The Shannon diversity index values for A_O_ and C_C_ at 0.03/0.05 distance were higher than A_C_ and C_O_, respectively, suggesting higher diversity in the microorganisms in A_O_ than A_C_, while similar higher diversity was observed in C_C_ when compare to C_O._ Since the anodes and cathodes of the two bioelectrochemical MBRs were initially inoculated with the same sludge, the observed differences in the microbial richness and diversity between the bioelectrodes could be attributed to power generation.

**Table 1:** Similarity based OTUs and species richness estimates of the bacterial phylotypes in the four samples.

	Cluster distance 0.03					Cluster distance 0.05				
	OUT	ACE	Chao1	Shannon	Coverage	OTU	ACE	Chao1	Shannon	Coverage
A_O_	1895	7816	4719	6.34	0.81	1602	5950	3780	6.07	0.85
A_C_	1488	5712	3424	5.73	0.87	1211	3846	2510	5.41	0.90
C_O_	1997	7981	4597	4.98	0.90	1583	4964	3143	4.64	0.93
C_C_	1856	8993	5204	5.63	0.82	1493	5443	3450	5.32	0.87

Note: Species richness was estimated using the program MOTHUR as described in Materials and Methods.

**Figure 2:**
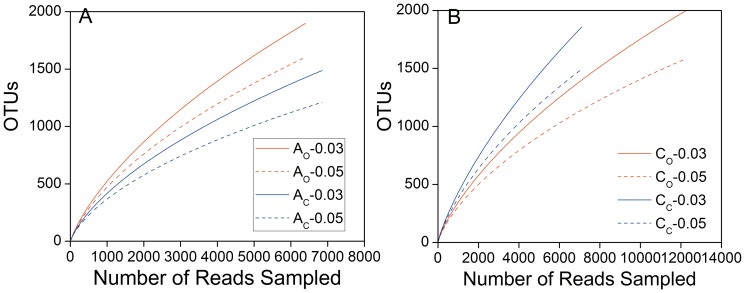
Rarefaction curves based on 16S rDNA sequences of the bacterial communities from anode (A) and cathode (B) samples. The OTUs are calculated based on 3% and 5% distances.

The rarefaction curves of all four samples at 3% and 5% distance thresholds are shown in Fig. 2. Evidently none of the rarefaction curves reached a plateau in this study, and new OTUs continued to emerge even after 12000 reads were sampled with pyrosequencing. The results indicated that pyrosequencing could successfully reveal the higher diversity of bacterial communities in MFC compared to other conventional molecular biological methods, such as DGGE and clone library [11]–[13]. Different slopes observed in the rarefaction curves represented diversity of the samples, and steeper slopes such as in A_O_ indicated higher sample diversity when compared to A_C_ and C_C_. Among all four samples C_O_ was the least diverse.

### Taxonomic complexity of the bacterial communities

**Figure 3:**
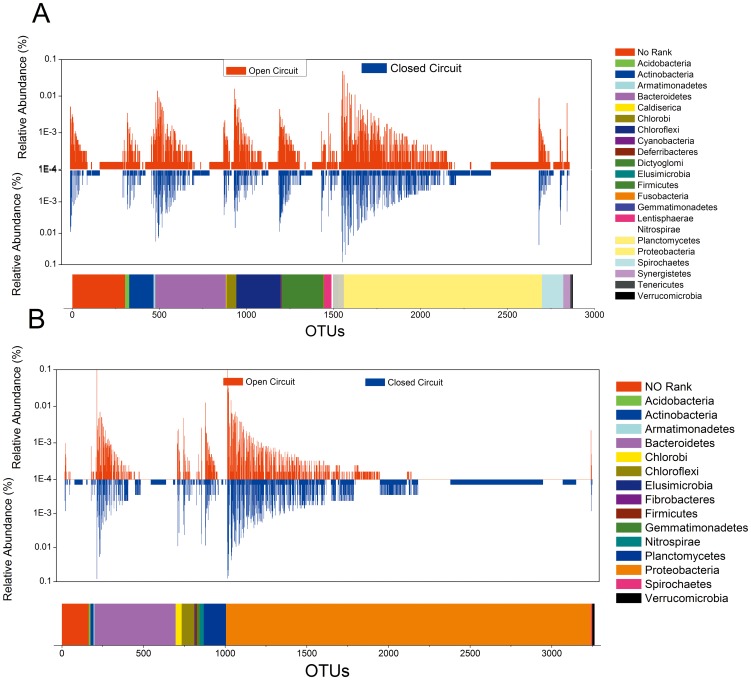
Relative abundance of bacterial reads retrieved from the anodes (A) and cathodes (B) in the open and closed circuit MFC models (Phylum level).

Phylogenetic analysis was used to characterize the anode and cathode microbial community structure and composition in the MBRs under open and closed circuit modes. A comparison of the relative bacterial community abundance at the phylum level for the anode and cathode samples is shown in Fig. 3. The number of phyla present in the A_O_ and A_C_ samples was nearly identical except for the minor phyla (*Deferribacteres* and *Gemmatimonadetes*), which accounted for less than 0.1% of the total community in the A_O_ sample.

In the anode samples, 22 phyla were identified. *Deferribacteres* and *Gemmatimonadetes* were detected in A_O_ while they were not present in A_C_. Among the total reads, 7.1% from A_O_ and 5.3% from A_C_ were not classified at the phylum level, indicating that at least some bacteria in the anode biofilms were not cultured. *Proteobacteria* and *Bacteroidetes* (45.2% and 15.5% for A_O_; 55.0% and 14.8% for A_C_) were the most abundant in both anode samples (Fig. 3), which is consistent with previous studies on MFCs anode biofilm analyzed by 454 pyrosequencing [14]–[16]. It should be noted that *Proteobacteria* was less abundant in A_O_ than in A_C_, which could be associated with the differential abundance in *Deltaproteobacteria* (25.1% in A_O_ and 38.9% in A_C_). The reason for this could be that some of the known exoelectrogenic bacteria (e.g., *Desulfuromonas*) belong to the phylum *Deltaproteobacteria* [17]. We also found that *Chloroflexi* (10.6%) was another major phylum represented in A_O_ sample. Previous studies had reported that the predominant bacteria in the anaerobic digester sludge was *Chloroflexi* [18], which is capable of assimilating N-acetyl-D-glucosamine, a major structural component in bacterial cells [19]. In the A_C_ sample, the third major phylum was *Firmicutes* (8.14%) besides the most abundant phyla *Proteobacteria* and *Bacteroidetes*. Previous studies have shown that *Firmicutes* was predominant in glucose-fed MFCs [16], [20], and the phylum could not be identified when MFC was switched to acetate-fed mode [20]. However, *Firmicutes* was detected in both anode samples in the present study, suggesting that *Firmicutes* could have originated from the inoculum and/or by symbiotic relationship among bacteria in the anodes. Moreover, *Firmicutes* was enriched in A_C_ than A_O_, which could be related to its ability to transfer extracellular electrons and it is likely that the metabolites produced by *Pseudomonas* (also enriched in A_C_) enabled *Firmicutes* (Gram-positive) to achieve this extracellular electron transfer function [21].

In the cathode samples, only 15 phyla were identified in total. *Fibrobacteres* were absent in C_O_, while *Armatimonadetes* and *Spirochaetes* were not found in C_C_. Only 1.08% of the total sequences in C_O_ and 1.06% in C_C_ were not identified at the phylum level, which were lesser than those in the anode samples. The dominant phyla were *Proteobacteria* (64.85% in C_O_ and 71.61% C_C_) and *Bacteroidetes* (25.2% in Co and 19.2% in C_C_) (Fig. 3), followed by *Planctomycetes* (4.75%) and *Chloroflexi* (1.45%) in C_O_, and *Planctomycetes* (2.92%) and *Chlorobi* (1.70%) in C_C_. Although the majority of phyla in both cathode samples were similar, C_O_ had abundant *Bacteroidetes*, while C_C_ had more *Protebacteria*, which comprised of *Alphaproteobacteria* (C_O_ - 5.7%, C_C_ - 15.3%), *Betaproteobacteria* (C_O_ - 49.2%, Cc - 45.3%), *Deltaproteobacteria* (C_O_ - 3.1%, Cc - 3.1%), *Gammaproteobacteria* (C_O_ - 6.1%, Cc - 6.4%), and *Epsilonproteobacteria* (C_O_ - 0.1%, Cc - 0.01%) classes. *Betaproteobacteria* is a well-known ammonia-oxidizing bacteria, and was discovered as a low abundant class in cathode biofilms from ammonia-lacking MFC [22], and the predominant class in ammonia-containing MFCs [14], [23]. The enrichment of *Betaproteobacteria* in both cathode samples was consistent with the high ammonia removal efficiency of MBR_C_ and MBR_O_ (97.5% and 96.4%). Further, *Alphaproteobacteria* was robust in C_C_ than C_O_, suggesting that the cathode in the bioelectrochemical MBR under closed-circuit operation facilitated the growth of *Alphaproteobacteria* [24]. In comparison with traditional dynamic MBR, the microbial community compositions in the bioelectrochemical MBR are also different. For instance, *Chlorobi*, *Chloroflexi* and *Planctomycetes* with relative abundance >1% were detected in our system, while they were absent in the dynamic MBRs reported by others [25]. This might indicate that the bioelectrochemical MBR owns its special community possibly due to the power generation.

### Changes in the microbial community in bioelectrochemical MBRs under open and closed circuit modes

**Figure 4:**
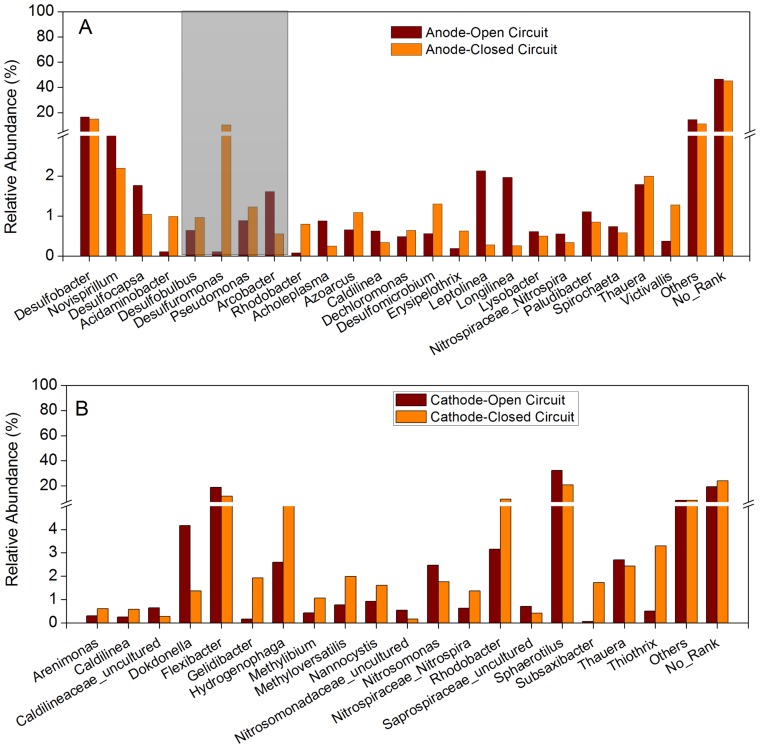
Relative abundance of the predominant groups in anode (A) and cathode (B) samples at the genus level. Relative abundance is defined as the number of sequences affiliated to a particular taxon divided by the total number of sequences per sample (%). Genera with relative abundance less than 0.5% in both libraries are defined as “others”.

To further elucidate the differences among microbial communities between the two systems, all four samples were compared at the genus level. Relative abundance (>0.5%) of the most dominant taxa in A_O_ and A_C_ is depicted in Fig. 4A. Interestingly, *Desulfobacter*, which has not been reported in any bioelectrochemical systems (BES) thus far, was the most abundant bacteria (16.6% in A_O_ and 15.0% in A_C_) in the anodes. This could be attributed to the fact that the inoculum used in this study was collected from the anaerobic unit of an anaerobic/anoxic/oxic-MBR with a longer sludge retention time (SRT = 60 d) and indeed, *Desulfobacter* was reported previously in this MBR [26]. Although both systems had the same dominant genus (*Desulfobacter*), the microbial communities between the two anode samples were quite different at the genus level. The relative abundance of *Desulfuromonas*, which is a known electrogenic bacteria, was much higher in A_C_ (10.2%) when compared to A_O_ (0.11%). This indicated that the power generation impacts microbial metabolism and that closed-circuit operation acclimated more electrogenic bacteria.

Microbial groups with electrochemical activities were observed in the anodes (gray shaded region in Fig. 4A) and included *Desulfobulbus*, *Desulfuromonas*, *Pseudomonas* and *Arcobacter* (0.64%, 0.11%, 0.89% and 1.6% in A_O_; 0.96%, 10.2%, 1.2% and 0.55% in A_C_, respectively). *Desulfobulbus* is reported to not only use soluble electron-acceptors such as sulfate and Fe (III) but also uses the electrode surface as electron acceptor when pyruvate, lactate, propionate or hydrogen are provided as electron donors without exogenous electron-shuttling compounds for electricity production [27]. *Desulfuromonas* can also oxidize ethanol, propanol, and butanol with Fe (III) as electron acceptor [28]. However, Bond et al. reported that *Desulfuromonas* can conserve energy to support their growth by oxidizing organic compounds such as acetate and benzoate with an electrode acceptor [29]. *Pseudomonas* can produce a soluble redox metabolite, pyocyanin, which mediates the transfer of electrons between bacteria and the anode in an MFC [30]. Besides, *Arcobacter* can associate with the electrode and rapidly generate a strong electronegative potential in the presence of acetate [31]. In the present study, the high abundance of *Desulfuromonas* and low abundance of *Desulfobulbus* and *Pseudomonas* is likely due to the use of acetate as electron donor. However, *Arcobacter*, which uses acetate as the electron donor and an electrode as the electron acceptor, was abundant in A_O_ than A_C_ presumably due to the competition between *Desulfuromonas* and *Arcobacter* for the electron donor (acetate). In addition to these, A_C_ also contained some minor bacterial populations (relative abundance <1%) such as *Clostridium*, *Comamonas* and *Geobacter* all of which have the capacity to generate electricity [17].

*Leptolinea* and *Longilinea* were represented in larger fractions in A_O_ (2.1% and 2.0%) than in A_C_ (0.28% and 0.26%) (Fig. 4A), suggesting that these two genera might not grow well in the closed-circuit anode biofilm. Such limitation in Ac could be due to the fact that both are strict filamentous anaerobes used in fermenting carbohydrates, and are in general isolated from thermophilic or mesophilic sludge [32]. Notably, *Rhodobacter*, a well-known photofermentative bacteria that produces hydrogen from many organic substrates [33], [34] was enriched in A_C_ (1.61%) than A_O_ (0.55%). Previously, *Rhodobacter* was shown to be directly involved in power production [35].

Interestingly, the microbial community structure between C_O_ and C_C_ samples was very different (Fig. 4B). *Sphaerotilus* was the most dominant bacteria in both C_O_ and C_C_ samples with a relative abundance of 32.2% and 20.8% respectively, followed by *Flexibacter* (18.9%), and *Dokdonella* (4.2%) in C_O_, and *Flexibacter* (11.7%), and *Rhodobacter* (9.3%) in C_C_. Both *Sphaerotilus* and *Flexibacter* were the dominant genera shared by both C_O_ and C_C_ samples, and these genera have been reported to be frequently responsible for filamentous bulking in activated sludge [36], [37]. However, in Cc a decrease in their abundance demonstrated that the closed-circuit conditions can alleviate filamentous bulking of activated sludge. *Rhodobacter*, which is a hydrogen producing bacteria [33] and *Hydrogenophaga*, an autotrophic H_2_-oxidizing bacteria that utilizes hydrogen as the energy source [38], were accumulated in the C_C_ biofilm (9.3% - *Rhodobacter* and 5.0% - *Hydrogenophaga*) (Fig. 4B). However, they only accounted for 3.2% and 2.6% in C_O,_ respectively. Thrash et al. demonstrated that microorganisms could accept electrons from a solid-state electrode via the cathodic production of hydrogen, which mediates the transfer of electrons [39]. Therefore, both *Rhodobacter* and *Hydrogenophaga* could be presumed to play a role in electron transfer from the cathode surface to the terminal electron acceptor.

Through 454 pyrosequencing, the functional microbes for bioelectricity generation are clarified. The present work also shows that the microbial community structure in the cathode is different. These results present the potential for further improving the system performance through regulation of electrogenic microbes as microorganisms play a key role in organic matter degradation and power generation. The enrichment of functional microbes for enhancing the power density needs further studying since now the optimization of the system mainly relies on reactor configuration, electrode materials and separators.

## Conclusions

High-throughput 454 pyrosequencing revealed that the bioelectrochemical MBRs operated under open and closed circuit conditions could result in diverse microbial community structures. Chao1 estimators and Shannon diversity indexes indicated that the microorganisms in A_O_ was richer and highly diverse than those in A_C._ However, among the cathodes C_C_ revealed a richer and more diverse microbial community when compared to C_O_. The microbial community composition in the anode samples revealed that *Proteobacteria* and *Bacteroidetes* were the dominant phyla in Ao and Ac, while *Firmicutes* was enriched in A_C_. *Deltaproteobacteria* affiliated to *Proteobacteria* were also more abundant in A_C_ than A_O_. In addition, the relative abundance of *Desulfuromonas*, which are well-known electrogenic bacteria, was much higher in A_C_ compared to A_O_, indicating that closed-circuit operation can acclimate more electrogenic bacteria. In the cathode samples, *Protebacteria* were robust in C_C_ while *Bacteroidetes* were more abundant in C_O_. *Rhodobacter* and *Hydrogenophaga* were represented more in C_C_ than C_O_, suggesting that the two genera could play a role in electron transfer from the surface of cathode to the terminal electron acceptors in the bioelectrochemical MBR under closed-circuit operation. Changes in the microbial community between the two bioelectrochemical MBRs demonstrated that the power generation affected the microbial community structure in both anode and cathode biofilms, facilitating the selection of functional microbes in the closed-circuit operation system for power generation.
